# On-chip photonic synapse

**DOI:** 10.1126/sciadv.1700160

**Published:** 2017-09-27

**Authors:** Zengguang Cheng, Carlos Ríos, Wolfram H. P. Pernice, C. David Wright, Harish Bhaskaran

**Affiliations:** 1 Department of Materials, University of Oxford, Parks Road, Oxford OX1 3PH, UK.; 2 Institute of Physics, University of Muenster, Heisenbergstrasse 11, 48149 Muenster, Germany.; 3 Department of Engineering, University of Exeter, Exeter EX4 QF, UK.

## Abstract

The search for new “neuromorphic computing” architectures that mimic the brain’s approach to simultaneous processing and storage of information is intense. Because, in real brains, neuronal synapses outnumber neurons by many orders of magnitude, the realization of hardware devices mimicking the functionality of a synapse is a first and essential step in such a search. We report the development of such a hardware synapse, implemented entirely in the optical domain via a photonic integrated-circuit approach. Using purely optical means brings the benefits of ultrafast operation speed, virtually unlimited bandwidth, and no electrical interconnect power losses. Our synapse uses phase-change materials combined with integrated silicon nitride waveguides. Crucially, we can randomly set the synaptic weight simply by varying the number of optical pulses sent down the waveguide, delivering an incredibly simple yet powerful approach that heralds systems with a continuously variable synaptic plasticity resembling the true analog nature of biological synapses.

## Teaser

An on-chip photonic device mimics the function of synaptic connections between neurons.

## INTRODUCTION

**Fig. 1:**
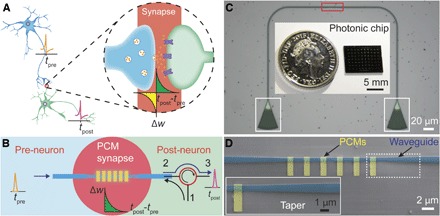
On-chip photonic synapse. (**A**) Structure of neuron and synapse. Inset: Illustration of the synapse junction. (**B**) Schematic of the integrated photonic synapse resembling the function of the neural synapse. The synapse is based on a tapered waveguide (dark blue) with discrete PCM islands on top, optically connecting the presynaptic (pre-neuron) and the postsynaptic (post-neuron) signals. The red open circle is a circulator with port 2 and port 3 connecting the synapse and the post-neuron; weighting pulses are applied through port 1 to the synapse. (**C**) Optical microscope image of a device with the active region (red box) as the photonic synapse. The optical input to and output from the device are via apodized diffraction couplers (white boxes). Inset: A typical photonic chip containing 70 photonic synapses has a dimension smaller than a 5-pence coin. (**D**) Scanning electron microscope image of the active region of the photonic synapse corresponding to the red box in (C) with six GST units (1 μm × 3 μm, yellow, false-colored) on top of the waveguide (blue, false-colored). Inset: The zoomed-in tapered structure of the waveguide highlighted by the white dashed box.

In stark contrast to conventional computing systems based on the von Neumann architecture where the central computing unit is separated from the main memory, the human brain contains a large number of neurons with synapses, each of them acting as both the computing and the memory unit (*1*). This unique structure makes the brain energy-efficient (*2*) in dealing with emotions, learning, and thinking—actions that are nearly impossible, or at least far less efficient and effective, for traditional computers (*3*). As shown in Fig. 1A, a neuron (pre-neuron) generates action potentials (spikes, fire time *t*_pre_) that propagate along the axon and are transmitted through a junction to the next neuron (post-neuron) that generates the postsynaptic action potentials (fire time *t*_post_). The junction is called a synapse (inset in Fig. 1A), with the synaptic weight (*w*) determining the communication strength between the two neurons. The synaptic plasticity (that is, the change in synaptic weight) Δ*w* is determined by neural activities, for example, Δ*w* = *f*(*t*_post_ − *t*_pre_) based on the Hebbian learning rule (*4*), and is believed to be the primary mechanism for memory and learning in the human and animal brain (*5*). Inspired by the brain, neuromorphic computing that attempts to imitate the neural system at the physical level has gained significant attention, recently driven by the needs of “big data” (*6*), artificial intelligence (*7*), and a supporting computing concept for the internet of things (*8*). As a first and key step to the construction of neuromorphic architecture, it is essential to develop suitably plastic synapse-like devices (*1*, *9*, *10*)—not least because synapses are by far the most numerous component of real brains, outnumbering neurons by several orders of magnitude (*2*).

Several electronic devices have recently been investigated to achieve synaptic function, such as those based on electrically induced resistive changes in phase-change chalcogenides (*11*–*13*), metal-insulator-metal structures (*14*, *15*), and ferroelectric materials (*16*), as well as nanomaterial-based field-effect transistors (*17*, *18*). A photonic synapse based on microfibers (*19*) and an optoelectronic synapse using carbon nanotubes (*20*) have also been demonstrated with potential benefits of large bandwidth (*21*, *22*) and no electrical interconnect power loss (*22*) inherently from the computing by optical means, although they tend to be either difficult to integrate and speed-limited, or still rely on electrical excitation signals. Here, we demonstrate a fully integrated all-photonic synapse based on phase-change materials (PCMs) that resembles the neural synapse at the physical level and can achieve synaptic plasticity compatible with the well-known Hebbian learning or spike timing–dependent plasticity (STDP) rule.

## RESULTS

### Concept of on-chip photonic synapse

The concept of a photonic synapse is shown schematically in Fig. 1B. A waveguide with discrete PCM structures on top acts as the photonic synapse with the input and output of the waveguide connected with a pre-neuron and a post-neuron. An optical circulator is used for connecting the output of the synapse and the post-neuron (from port 2 to port 3) and for applying optical pulses to alter the synaptic weight (from port 1 to port 2). Low-energy optical transmission can be measured from the pre-neuron to the post-neuron, with the transmission level dependent on the synaptic weight.

It has previously been demonstrated that optical pulses can switch PCMs integrated on waveguides to provide non-volatile photonic memories storing up to eight levels in a single cell (*23*). To move between levels in that memory implementation required single pulses with varying powers for amorphization and a complicated multipulse, multipower format for recrystallization. However, for the realization of a practicable synapse mimic, not only precise control of synaptic weighting but also a device whose weight is controlled by fixed pulse characteristics is crucial. Here, we achieve this by designing a tapered waveguide structure and then incorporating multiple, small, discrete PCM islands (Fig. 1, C and D). This enabled us to have much improved control of the electric field (of the optical pulses propagating along the waveguide) that interacts with the PCM. This resulted in a very effective method for synaptic weight control that is based entirely on the number of fixed-duration, fixed-power excitation pulses applied, as we demonstrate later.

### Synaptic-mimic design with FEM analysis

We use the well-studied chalcogenide Ge_2_Sb_2_Te_5_ (GST) (*24*–*27*) for the PCM cells, with indium tin oxide (ITO) as the capping layer (see Materials and Methods). Figure 1C shows the optical image of a typical device. Two diffraction grating couplers are used to connect the device with fiber arrays for signal transmission (Fig. 1C and fig. S1). The central part of the waveguide (with the discrete GST islands on top) is tapered to a 0.8-μm width from a nominal width of 1.3 μm elsewhere (Fig. 1D).

**Fig. 2:**
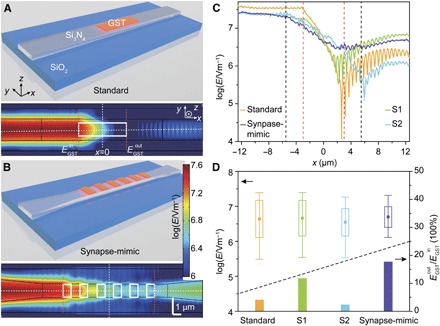
Finite element method (FEM) simulations of the photonic synapse with different structures. (**A**) Top: Schematic shows photonic synapse with a standard design: a straight waveguide with a thin film of GST (6 μm × 0.8 μm, orange block) on top. Bottom: TE mode *E*-field distribution at the surface of the waveguide with the entire GST film (white box) in the crystalline state. (**B**) Top: Schematic shows photonic synapse with synapse-mimic design: a tapered waveguide with six discrete GST islands (1 μm × 0.8 μm each) on top, which is the structure used in our experiments. Bottom: *E*-field distribution with all GST islands in crystalline states. (**C**) *E*-field distributions along the center line of the waveguide surface. The yellow, purple, green, and cyan curves correspond to the *E*-field distribution along the dashed horizontal lines in (A) and (B) (standard and synapse-mimic) and fig. S3 (A and B) (S1 and S2), respectively. The dashed red (black) lines illustrate the left and right boundaries of the GST film (discrete GST islands) in standard and S1 (synapse-mimic and S2) designs. (**D**) Top: Statistical results of the *E*-field inside the GST film or islands (square, average value; box, SD; bottom and top lines, minimum and maximum values). Bottom: The ratio between the average *E*-field at the output ($E_{\text{GST}}^{\text{out}}$) and input ($E_{\text{GST}}^{\text{in}}$) edges of the GST film or islands corresponding to the orange dashed lines in (A) and (B) and fig. S3 (A and B).

The effectiveness of the combination of the tapered structure with the discrete PCM islands for controlling the interaction field along the waveguide is exemplified in Fig. 2 (and fig. S3). Here, a transverse electric (TE) optical field at 1580 nm is “launched” into the left side of several alternative waveguide designs (Fig. 2, A and B, and fig. S3, A and B), and the strength of the electric field (*E*-field) at the waveguide surface is simulated for the PCM in the crystalline (Fig. 2 and fig. S3) and amorphous phase (fig. S3). With the GST in the crystalline phase, a simple rectangular waveguide with a single large GST cell on top [Fig. 2A, as used in the memory application of Ríos *et al*. (*23*)], the *E*-field decays rapidly because of the very strong absorption and exhibits many resonant peaks and troughs (standard design in Fig. 2C). However, for the tapered waveguide with discrete GST islands (synapse-mimic design in Fig. 2C), the interaction of the *E*-field (and so the absorption) is much more “controlled,” with a much more gradual decay and little evidence of resonance effects. To quantitatively compare the electric field distributions in the various structures, we calculated the average, SD, and range of the *E*-field inside the GST regions, as well as the ratio between the fields at the right ($E_{\text{GST}}^{\text{out}}$) and left sides ($E_{\text{GST}}^{\text{in}}$) of the GST cells (Fig. 2D). The SD and the range of the electric field in the GST regions of the synapse-mimic structure are the smallest, and this structure also has the highest $E_{\text{GST}}^{\text{out}}/E_{\text{GST}}^{\text{in}}$ ratio, thus demonstrating the smoothest distribution of the *E*-field inside and that most energy transmitted past the GST cells in this structure (detailed comparisons between four structures are elucidated in section S2 of Supplementary Text).

### Synaptic weighting and plasticity

**Fig. 3:**
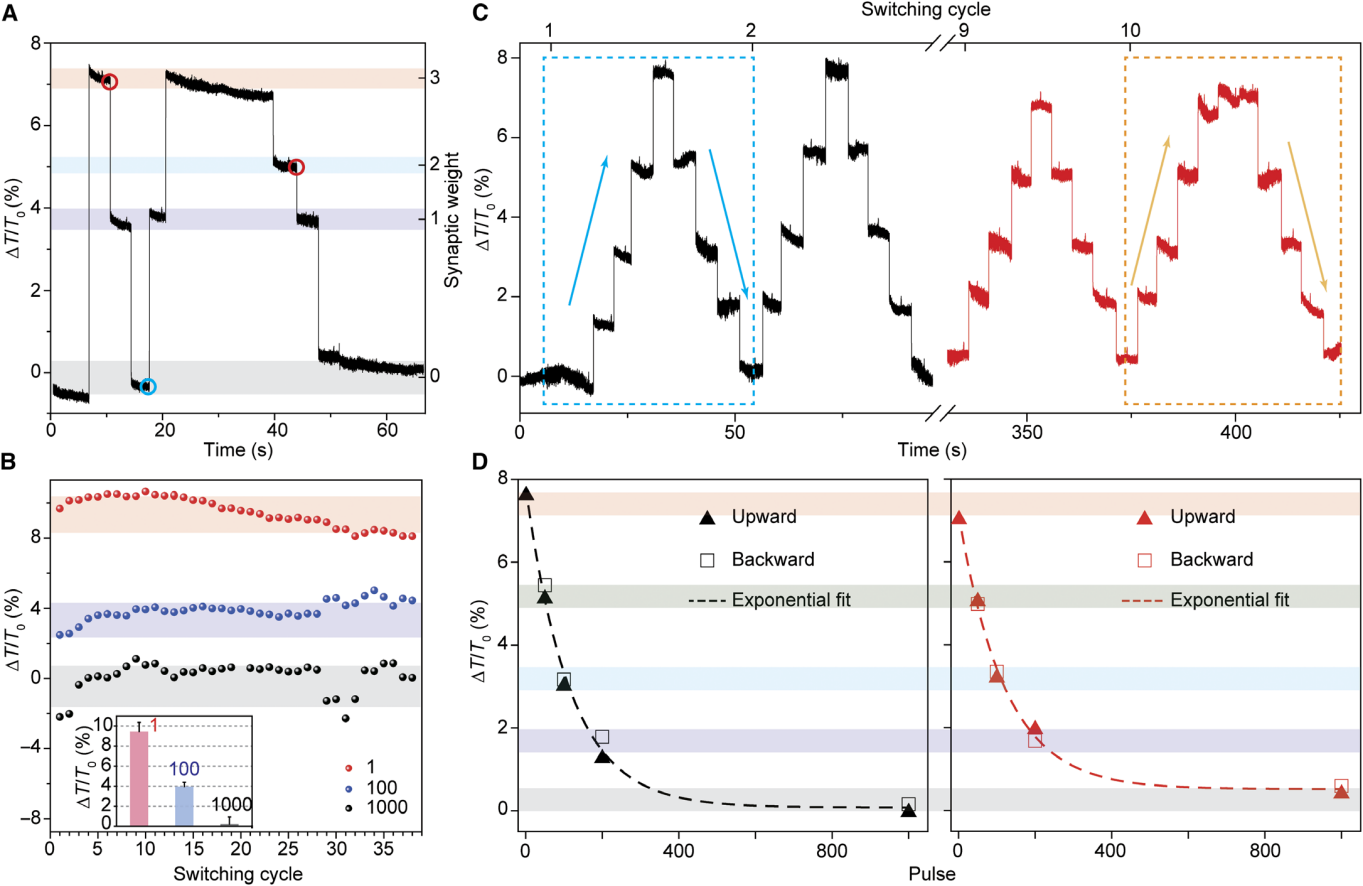
Synaptic weighting and plasticity. (**A**) Demonstration of differential synaptic weighting of the device in Fig. 1 during switching between the crystalline and amorphous states of GST islands with the relative transmission change (Δ*T*/*T*_0_) recorded. Each weight can be reached with the same number of pulses (50 ns at 243 pJ, 1 MHz) from any previous weight. (**B**) Repeatability of the weighting over multiple cycles. Inset: Statistical analysis of the change in readout for weight “0,” “1,” and “4.” The pulse applied here was 50 ns at 320 pJ, slightly larger than that in (A). (**C**) Five weights of the photonic synapse are obtained with switching at optical pulse energy (404.5 pJ, 50 ns). The dashed blue (yellow) boxes correspond to the first (last) weighting cycle. The upward and downward arrows in the boxes are the weighting directions. (**D**) Photonic synaptic weight (Δ*T*/*T*_0_) as a function of optical pulse numbers. The left (right) panel corresponds to the data of the dashed blue (yellow) box in (C). The solid triangles (hollow boxes) are the data from the upward (downward) weighting direction. The dashed lines are the exponential fittings of the data with detailed fitting parameters elucidated in table S2.

The enhanced *E*-field control engendered by the use of the structure comprising a tapered waveguide with discrete PCM islands leads directly to the implementation of a simple yet most effective integrated photonic synapse, as we now show. Before optical measurements, the devices were annealed on a hot plate (~250°C) for 10 min to completely crystallize the GST. The optical transmission (*T*_0_) of the device with the GST in the fully crystalline state is defined as the baseline of the readout and assigned to a synaptic weight “0.” Any subsequent change of the readout (Δ*T* = *T* − *T*_0_) during the measurement is normalized as the relative change in percentage (Δ*T*/*T*_0_) to the baseline (Fig. 3A). Changes in synaptic weighting, as shown in Fig. 3, were then achieved by sending fixed-duration, fixed-energy optical pulses down the waveguide. Using one single pulse of 50 ns at 243 pJ (section S1 of Supplementary Text), the transmission readout changed by ~7% to weight “3” (arbitrarily defined, but predetermined weight numbers). The weight could then be decreased from weight “3” to “1” with 100 identical pulses (repetition rate, 1 MHz; total weighting time, 100 μs) using the exact same pulse parameters (that is, 50 ns, 243 pJ). When we increased the pulse number to 1000 (total weighting time, 1 ms), the device went from weight “1” to “0.” This represents a crucial advance, because it allows one to arbitrarily adjust the synaptic weight using a set of known pulses without having previous knowledge of the current actual weight.

This finding is worth discussing further. For example, weight “1” in Fig. 3A can be switched from weight “3,” “2,” or “0” with exactly the same parameters of pulses (100 pulses of 243 pJ and 50-ns length); similarly, weight “0,” “2,” and “3” can be set with 1000, 50, and 1 pulses, respectively, from any (undetermined) previous weight. Moreover, we examined the long-term durability of the switching between different weights, as shown for example in Fig. 3B. Even after 38 cycles of switching, carried out over a period of many minutes, the individual weights are clearly distinguishable with the deviation of each weight below 0.77% in readout transmission. These results illustrate a direct and unambiguous achievement of deterministic synaptic weighting using a known number of optical pulses (section S3 of Supplementary Text).

To uncover the relationship between the synaptic weight and the pulse number in more detail, we increased the power of the pulse to obtain five stable weights with high signal-to-noise ratio (Fig. 3C and fig. S5). The device started at weight “0” (baseline), which was then followed by pulses of 200, 100, 50, and 1 (404.5 pJ, 50 ns) to reach weights “1,” “2,” “3,” and “4,” respectively; the pulse sequence was then reversed (that is, 50, 100, 200, and 1000 pulses applied corresponding to a total weighting update time of 50 μs, 100 μs, 200 μs, and 1 ms, respectively) to access weights “3,” “2,” “1,” and “0,” and the whole process was repeated 10 times. We extracted the mean value of the transmission change for each weight (monitored for ~10 s) from the 1st and 10th weighting cycle and plotted them versus the corresponding pulse numbers, shown in Fig. 3D. First, as previously stated, we see that the change in synaptic weight is exponentially and monotonically dependent on the number of pulses applied. This relation is very stable and is sustained for long periods of time (it took us 7 min to complete the experiments in Fig. 3C and fig. S5, for example). Moreover, we note that, by tuning of the pulse parameters, we can readily achieve an increased number of synaptic weights. For example, with a decreased pulse width of 20 ns and a pulse energy of 216 pJ, we readily achieved 11 synaptic weights, with a similar exponential relationship between the change of synaptic weight and the number of pulses (fig. S6). By further pulse control, and/or by increasing the signal-to-noise ratio [for example, by increasing the readout power (*23*)] and/or by further device optimization, it will be possible to access considerably more synaptic weighting levels or even achieve continuous weighting to mimic the true analog nature of synaptic weight change in biological systems (*28*).

### All-optical STDP plasticity

**Fig. 4:**
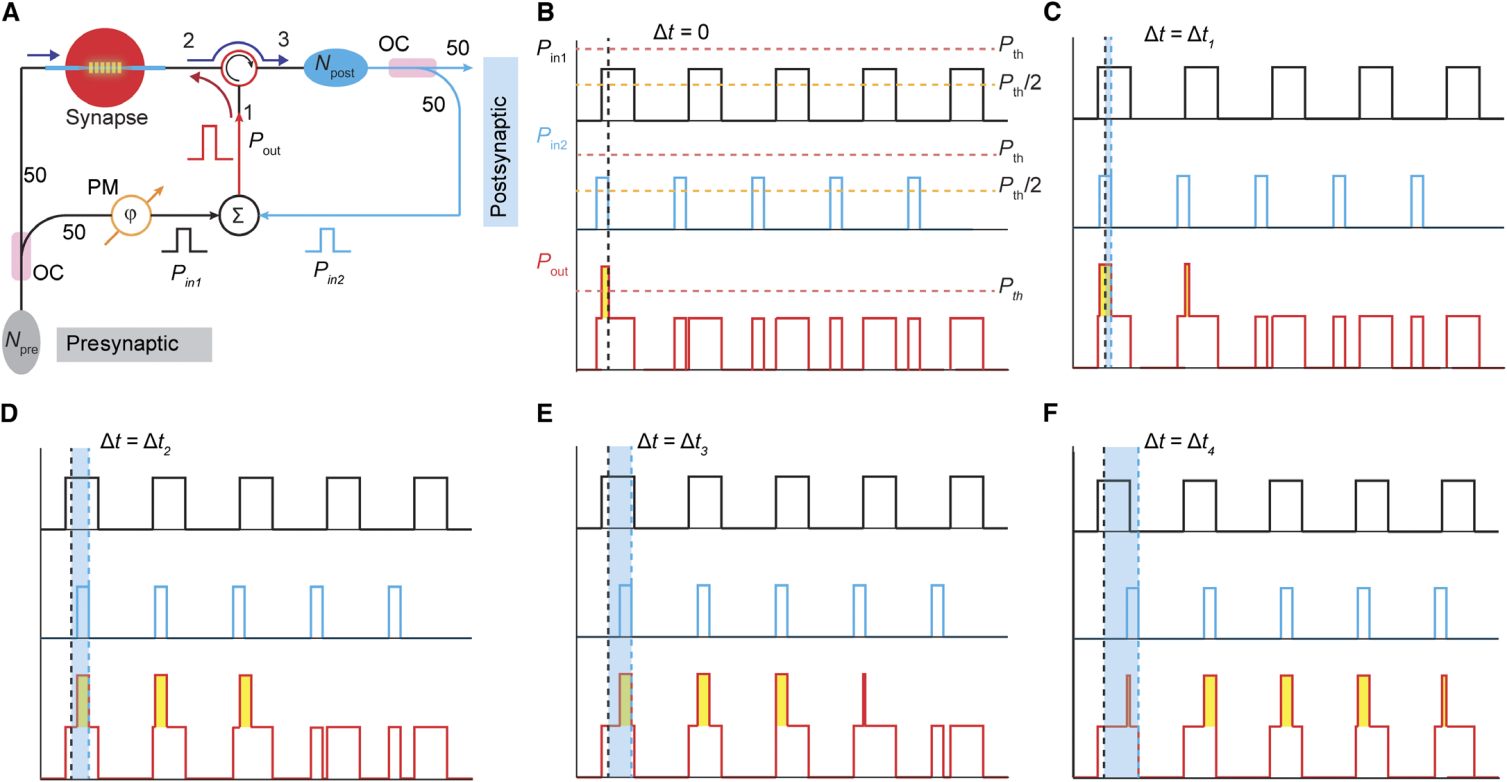
Proposed STDP scheme based on a photonic synapse. (**A**) Schematic of the all-optical method using a photonic synapse to achieve the STDP plasticity. Split with an optical coupler (OC) (50:50), 50% of the presynaptic signal is connected to one input (*P*_in1_) of an interferometer via a phase modulator (PM). Similarly, 50% of the postsynaptic signal is connected to the other input (*P*_in2_) of the interferometer. The output signal (*P*_out_) of the interferometer is used to update the synaptic weight. *N*_pre_ and *N*_post_ are pre- and postsynaptic neurons, respectively. (**B**) Illustration of presynaptic (black) and postsynaptic (blue) signals with no time delay (Δ*t* = 0) and the net output power of the interferometer as the switching signal (red). (**C** to **F**) The time delay between pre- and postsynaptic signals is increasing to arbitrarily chosen values Δ*t*_1_, Δ*t*_2_, Δ*t*_3_, and Δ*t*_4_, resulting in different numbers of pulses above the threshold switching power (*P*_th_) being sent to the synapse.

Finally, we point out that the exponential dependence of synaptic weight on the number of applied pulses in our photonic synapse leads to a very simple and compact implementation of the STDP rule using photonic integrated-circuit techniques. The STDP rule to describe synaptic weight change in a biological system has the form (*4*) of Δ*w* = *Ae*^−Δ*t*/τ^. Here, Δ*w* and Δ*t* are the synaptic weight change and the time delay between pre- and postsynaptic signals, whereas *A* and τ are constants. This STDP behavior could be achieved quite simply with an all-optical structure based on our photonic synapse (Fig. 4). As shown in Fig. 4A, the presynaptic signal with power *P*_pre_ is split into two beams with 50% coupled into a photonic synapse similar to that of Fig. 1, and the other 50% (*P*_in1_) is connected to an interferometer via a phase modulator. The postsynaptic signal (*P*_post_) is also divided into two parts, with 50% transmitted and the remainder (*P*_in2_) fed back to the interferometer. By adjusting the phase modulator, the net output power (*P*_out_) of the interferometer obtains the tunability between zero and (*P*_in1_ + *P*_in2_), and this output is used to update the weight of the synapse. In this particular design, the powers of pre- and postsynaptic signals are chosen to lie between *P*_th_ and 2 × *P*_th_ [where *P*_th_ is the threshold power for switching PCMs (*23*)] such that *P*_th_/2 < *P*_in1_, *P*_in2_ < *P*_th_, as shown in Fig. 4B. The pulse widths and repetition rates of pre- and postsynaptic signals are intentionally set differently. When there is no time delay (Δ*t* = 0) between the pre- and postsynaptic signals, the net output power from the interferometer applied to the synapse (the red trace in Fig. 4B) has a single pulse larger than *P*_th_. With increasing the time delay (Δ*t* = Δ*t*_1_, Δ*t*_2_, Δ*t*_3_, and Δ*t*_4_, arbitrarily chosen values), the number of output pulses with net power above *P*_th_ gradually increases to 2, 3, 4, and 5 in this example, as shown in Fig. 4 (C to F). By an appropriate design of the pre- and postsynaptic signals, the number of output pulses with power larger than *P*_th_ could be linearly dependent on Δ*t*, leading to the required exponential dependence of the synaptic weight change on the time delay (between pre- and post-neuron firings), thus mimicking the STDP behavior in a simple and effective manner.

## DISCUSSION

In conclusion, with a specially designed structure of discrete phase-change islands on a tapered waveguide, we have obtained a brain-inspired, on-chip biomimetic photonic synapse with analog and cumulative programmability, essential requirements (*29*) for neuromorphic computing. Optical field simulations demonstrate that the distribution of the electric field in the photonic synapse is much more homogeneous than in conventional waveguide designs. This feature allows for the deterministic adjustment of synaptic weights with a predetermined number of identical, fixed-energy, fixed-duration pulses. Furthermore, by intentionally arranging the pre- and post-neuron signals, we have elucidated an all-optical method to modulate the synaptic weight based on the time delay between the pre- and post-neuron signals that can mimic the STDP rule in biological systems. Moreover, via the use of improved/optimized device designs and switching protocols, along with the use of alternative PCMs having lower switching powers (cf. GST), it should ultimately be possible to realize large-scale photonic neuromorphic networks similar in scale to state-of-the-art electronic neuromorphic computers [for example, the SpiNNaker machine (*30*)] but operating at powers approaching that of the human brain (see section S4 of Supplementary Text). Significantly advancing our earlier work on accumulation-based computing (*31*) and integrated photonic memory (*23*, *27*) using PCMs, our study has established a novel architecture combining neuromorphic and photonic computing, with the synapse based on PCMs being the first crucial step. Future work might focus on the implementation of an on-chip “integrate and fire” neuron, which would complete the building blocks required to enable truly integrated, biologically inspired photonic computing paradigms.

## MATERIALS AND METHODS

### Device fabrication and characterization

The photonic synapses were fabricated on a Si_3_N_4_/SiO_2_ platform, as reported previously (*23*, *27*). A JEOL JBX-5500ZX 50-kV electron beam lithography system was used to define the photonic devices on the wafer spin-coated with Ma-N 2403 negative-tone resist, followed by reactive ion etching (PlasmaPro 80, Oxford Instruments) in CHF_3_/O_2_/Ar to etch down 300 nm of Si_3_N_4_. A second step of electron beam lithography using poly(methyl methacrylate)–positive resist was used to define the pattern of discrete PCM islands, and 10-nm GST/10-nm ITO was subsequently sputter-deposited. The structure of the photonic synapse was characterized by a scanning electron microscope (Hitachi S-4300) with a low accelerating voltage (1 to 3 kV). The images were obtained using the secondary electron detector at a working distance of ~13 mm.

### Finite element method simulations

The finite element method simulations were carried using the COMSOL Multiphysics software with the RF module. A TE mode optical field with a nominal power of 1 W was injected into the waveguide. The electromagnetic field distribution in the frequency domain was simulated inside a three-dimensional model of the waveguide with GST films or islands. The results shown in the text are the amplitudes of the electric fields recorded in the central cross section in the *x*-*y* plane of the structure.

### Optical measurement

Real-time transmission measurements of the photonic synapse during optical pulse switching were performed using a probe-pump technique, as described previously (*23*) and in section S4 of Supplementary Materials and Methods. The measurement setup is illustrated in fig. S2. Briefly, a low-power probe laser and a high-power pump laser working at different wavelengths were routed through the photonic synapse from opposite directions. Two optical circulators were used to guide one laser into the device while directing the other laser out to detectors. To suppress interference between the two signals, we used two optical band-pass filters (OTF-320, Santec) in the probe and pump lines. A continuous-wave (CW) diode laser (TSL-550, Santec) as a probe laser was used to interrogate the transmission of the photonic synapse. The pump pulse was generated from a CW diode laser (N7711A, Keysight) combined with an electro-optic modulator (EOM) (2623NA, Lucent) that was controlled by an electrical pulse generator (AFG 3102C, Tektronix), and subsequently amplified by a low-noise erbium-doped fiber amplifier (AEDFA-CL-23, Amonics).

## Supplementary Material

http://advances.sciencemag.org/cgi/content/full/3/9/e1700160/DC1

## SUPPLEMENTARY MATERIALS

Supplementary material for this article is available at http://advances.sciencemag.org/cgi/content/full/3/9/e1700160/DC1 Supplementary Materials and Methods Supplementary Text fig. S1. Design of photonic synapse. fig. S2. Optical measurement scheme. fig. S3. Optical field distribution in photonic synapses. fig. S4. Optical field distributions in S2 and synapse-mimic designs. fig. S5. Full trace of five-level weighting. fig. S6. Eleven-level weighting. table S1. Dimensions of photonic synapses. table S2. Fitting of synaptic weight on pulse number. References (*32*–*34*)
