# Supplementary material for: On-chip photonic synapse
Source: Sci Adv. 2017 Sep 27;3(9):e1700160. doi: 10.1126/sciadv.1700160 (PMC5617375; doi:10.1126/sciadv.1700160)
Supplement: http://advances.sciencemag.org/cgi/content/full/3/9/e1700160/DC1 [file supp_3_9_e1700160__index.html]

Science Advances | Science Advances

## Supplementary Materials

**This PDF file includes:**

- Supplementary Materials and Methods
- Supplementary Text
- fig. S1. Design of photonic synapse.
- fig. S2. Optical measurement scheme.
- fig. S3. Optical field distribution in photonic synapses.
- fig. S4. Optical field distributions in S2 and synapse-mimic designs.
- fig. S5. Full trace of five-level weighting.
- fig. S6. Eleven-level weighting.
- table S1. Dimensions of photonic synapses.
- table S2. Fitting of synaptic weight on pulse number.
- References (*32–34*)

Download PDF

**Files in this Data Supplement:**

- Adobe PDF - 1700160\_SM.pdf
