# Supplementary material for: On-chip photonic synapse
Source: Sci Adv. 2017 Sep 27;3(9):e1700160. doi: 10.1126/sciadv.1700160 (PMC5617375; doi:10.1126/sciadv.1700160)
Supplement: http://advances.sciencemag.org/cgi/content/full/3/9/e1700160/DC1 [file 1700160_SM.pdf]

Science Advances

AAAS

advances.sciencemag.org/cgi/content/full/3/9/e1700160/DC1

Supplementary Materials for

On-chip photonic synapse

Zengguang Cheng, Carlos Ríos, Wolfram H. P. Pernice, C. David Wright, Harish Bhaskaran

Published 27 September 2017, *Sci. Adv.* **3**, e1700160 (2017)
DOI: 10.1126/sciadv.1700160

This PDF file includes:

- Supplementary Materials and Methods
- Supplementary Text
- fig. S1. Design of photonic synapse.
- fig. S2. Optical measurement scheme.
- fig. S3. Optical field distribution in photonic synapses.
- fig. S4. Optical field distributions in S2 and synapse-mimic designs.
- fig. S5. Full trace of five-level weighting.
- fig. S6. Eleven-level weighting.
- table S1. Dimensions of photonic synapses.
- table S2. Fitting of synaptic weight on pulse number.
- References (*32–34*)

Supplementary Materials and Methods

1. Photonic synapse design

The overall structure and relevant parameters of photonic synapse design are shown in fig. S1. Briefly, the parameters for the waveguide are as follows: H is the total length of the vertical straight part of the waveguide; L is the total length of the horizontal straight part of the waveguide; r is the radius of the curved regions of the waveguides on the left and right.

The zoomed part of the active region (dashed black box in fig. S1A) of the photonic synapse is shown in fig. S1B with the parameters as: L_1 is the total length of the active region; L_2 is the length of the central part with the smallest width w_c where several pieces of PCMs are distributed equally in that region; w_g and p are the width of each PCM and the spacing between them respectively; w_{lr} is the width of the waveguide at the edges of the active region larger than w_c. From the edges to the central part, the width linearly decreases from w_{lr} to w_c in the transition region.

The detailed structure of the apodized grating coupler (dashed blue box in fig. S1A) is illustrated in fig. S1C with three regions shadowed as I (orange), II (yellow) and III (blue). Region I: the transition part of the coupler. This part is a sector structure with the angle of α and a flat tip at the end whose width is also w_{lr}. The length of region I is tl. Region II: this part has ten concentric arcs that have the same center with the sector in region I. The pitch is fixed as Λ while the width of each arc a_1 is linearly decreasing from the first to the tenth which makes this part the appodized grating region. Region III: the last part of the coupler with uniform gratings. It has similar arc structures as Region II with both pitch Λ and width a_0 fixed. The specific parameters for the optical synapse used in our experiments are summarized in table S1.

2. Device fabrication

The fabrication process of the photonic synapse is based on methods described previously (*23*). The key steps are as follows: (i) The silicon nitride wafer (330 nm Si_3N_4/3.3 μm SiO_2, Rogue Valley Microdevices, Medford, OR, USA) was cleaned by acetone,

isopropanol and deionized water in sequence and baked (150°C/10 min). (ii) The substrate was spin-coated with Ti-prime (Microchemicals GmbH, Ulm, Germany) and ma-N 2403 (micro resist technology GmbH, Berlin, Germany) double layer resist, prebaked (120°C/2 min, 90°C/2 min respectively), and then (iii) patterned by E-beam lithography (JEOL 5500FS) at 50 kV to define the bottom waveguide structure. (iv) After developing in MF-319 developer (Dow Chemical Company) for 1 min, the waveguide pattern was post baked (100°C/2 min) to reduce the surface roughness. (v) Subsequently, reactive ion etching in $CHF_3/O_2/Ar$ was used to etch down 300 nm of Si_3N_4, followed by the complete removal of the remaining resist by Remover mr-Rem 660 (micro resist technology GmbH). (vi) The second e-beam lithography process was used to pattern GST/ITO elements. Briefly, the substrate was spin-coated with double layer of 495 PMMA at 4% and 950 PMMA at 2% (MicroChem Corp.) and pre-baked at 150°C for 5 min and 15 min respectively. The pattern was defined by e-beam lithography followed by developing in IPA/MIBK/EMK (15:5:1; MicroChem Corp.). (vii) 10 nm GST/10 nm ITO were subsequently sputtered on the waveguide using a Nordiko RF sputtering system (Nordiko Technical Services Ltd.). (viii) Finally, the liftoff process for the GST/ITO layers was carried out in Remover PG (Micro Chem Corp.).

3. Scanning electron microscope (SEM) characterization

The structure of the photonic synapse was characterized by SEM (Hitachi S-4300) with low accelerating voltage (1 to 3 kV). The images were obtained by secondary electron detector at a working distance of ~13 mm.

4. Optical measurement setup

The optical measurement is similar to the setup described previously (*23*) illustrated in fig. S2. Two continuous-wave (CW) diode lasers with separated wavelengths (probe laser: λ_{probe} = 1570 nm, purple; pump laser: λ_{pump} = 1580 nm, red) are used to achieve on-chip probe/pump measurements. The probe laser (TSL-550, Santec Europe Limited, London, UK) operates in CW mode at low-power (1 mW) to interrogate the transmission change of the photonic synapse. The pump laser (N7711A, Keysight Technologies) is

modulated by an electro-optical modulator (2623 NA, Lucent Technologies) which is controlled by an electrical pulse generator (AFG 3102C, Tektronix). Subsequently, the optical pulse is amplified by a low-noise erbium-doped fiber amplifier (AEDFA-CL-23, Amonics Ltd.). To monitor the transmission response of the optical synapse when excited by an intense pump pulse, a bidirectional scheme was used with probe and pump lasers guided through the device from opposite directions (Left: probe and Right: pump in fig. S2). In detail, two optical circulators (6015-3-APC, Thorlabs) are connected to the probe and pump signals respectively and each circulator can direct one optical signal into the device (from port 1 to port 2) and out from the device (from port 2 to port 3). To eliminate any reflection inside the device and make the probe and pump routes separated from each other, two optical band-pass filters (OTF-320, Santec Europe Limited) with bandwidth of ~0.35 nm at -3 dB are used in the probe and pump paths with resonant wavelengths λ_{probe} and λ_{pump} respectively. In addition, three polarization controllers (FPC032, Thorlabs) are used in the input path from both directions to optimize the transmission of the optical synapse. The optical signals are coupled into the integrated optical chip mounted on a 3-axis motorized piezo stage via an optical fiber array that are aligned with the appodized grating couplers of the device. The pump signals after propagation through the photonic synapse is detected by a fast 1 GHz photoreceiver (1611C-AC, Newport Spectra-Physics Ltd.) connected to an oscilloscope. The probe signal propagated from the optical chip splits into two paths via an optical coupler (10202A-90-APC, Thorlabs) with 90% going to a fast 125 MHz photoreceiver (1811-FC, Newport Spectra-Physics Ltd.) and subsequently connects to the oscilloscope while 10% goes to a 200 kHz photoreceiver (2011-FC, Newport Spectra-Physics, Ltd.) that is recorded using a data acquisition (DAQ) card (USB-6009, National Instruments Corporation (UK) Ltd.) connected to a computer.

Supplementary Text

1. Optical pulse switching and weighting

In order to obtain an optical pulse, the pump laser is modulated by the electro-optical modulator (EOM) that is controlled using an electrical pulse generator. The optical pulse has the same pulse width and frequency as the electrical modulation pulse. Briefly the parameters for the electrical pulse generated from the pulse generator are as follows: Frequency: 1 MHz; Voltage high level: 2 V; Voltage low level: -1 V; Pulse width τ_{pulse}: 20 ns (fig. S6) and 50 ns (other cases); Rise and fall edge: 5 ns.

The output power of the CW pump laser is 4 mW that is modulated by the EOM (Extinction ratio: 13 dB) and amplified by the EDFA. The pulse signal from the EDFA P_{amp} is further suppressed by the OTF and the coupling loss from the fiber array to the waveguide. The optical power P_{GST} in the waveguide before the PCM is calculated as: $P_{GST} = P_{amp} \times r_{filter} \times r_{coupler}$, where r_{filter} and $r_{coupler}$ are the attenuation ratio of the OTF and the loss ratio from the coupling of fiber array with the waveguide, respectively. The pulse energy E_{GST} in our study is calculated as: $E_{GST} = P_{GST} \times \tau_{pulse}$.

During the weighting process, a single optical pulse or fixed number of optical pulses with 1 MHz repetition rate (limited by the pulse generator) were used to reach different synapse weights. For a weight accessed by a single optical pulse, the weighting time is determined by the pulse width applied, such as 50 ns weighting time of the weight '3' in Fig. 3 and fig. S5, and 20 ns weighting time of the weight '10' in fig. S6. The weighting time of multi-pulses is dominated by the pulse generator's repetition rate (1 MHz) equivalent to 1 μs in time, therefore the weights '2' (50 pulses), '1' (100 pulses) and '0' (1000 pulses) in Fig. 3A for example have the weighting time of 50 μs, 100 μs and 1 ms respectively (and similar weighting update times apply for results shown in fig. S5, fig. S6 etc.). As picosecond optical pulses (1 ps at 40 MHz repetition rate) have been shown capable of successfully switching phase change cells on integrated optical waveguide (*32*), there is the promise of reaching significantly faster weighting speeds for the photonic synapse of our manuscript by using such picosecond laser sources and/or fast pulse generators. Furthermore, it should be noted that after sending weighting pulses to

the photonic synapse, each obtained weight (optical transmission) was recorded for a duration of around 5 to 20 seconds, so as to allow for a proper statistical analysis of each weight (and it is this recording 'dwell' time that accounts for the long overall weighting cycle times of Fig. 3, A and C, fig. S5 etc.).

In terms of the endurance of the synaptic devices (i.e. the number of times they can switched between amorphous and crystalline states), we note that a pulse of 50 ns at 320 pJ was applied to reach 38 weighting cycles in Fig. 3B of the main text. After the 38th cycle the device was still operational, it was just that testing ceased and it is noted that these phase-change materials are proven for endurance of at least 10^{11} switching cycles (*33*), meaning that there is unlikely to be any inherent materials limitation to prevent the achievement of high endurance in our devices.

2. FEM simulations and discussion

The FEM simulations were carried using COMSOL Multiphysics® software incorporating the RF module. A TE mode optical field at 1580 nm with a power of 1 W was simulated. The electromagnetic wave in the frequency domain was simulated inside the 3D model of the waveguide with the GST film or islands. The results shown in the Main Text and Supplementary Materials are the amplitudes of the electric fields distributed in the central cross-section of the structures perpendicular to the *z* direction.

Four distinct photonic synapses with variant combinations of the waveguide structure and the GST form were simulated as: straight waveguide with thin film of GST (Standard design, Fig. 2A), tapered waveguide with thin film of GST (S1, fig. S3A), straight waveguide with discrete islands of GST (S2, fig. S3B) and tapered waveguide with discrete islands of GST (Synapse-mimic design, Fig. 2B).

i) In the Standard photonic synapse, there was a substantial light absorption in crystalline GST (Fig .2A) with little effect when the GST is amorphous (Standard of fig. S3C);

ii) Subsequently the waveguide was shrunk to a tapered structure (S1). For the amorphous GST film (S1 of fig. S3C), the output (right edge) of S1 shows some transmission loss with the electric field extending outside of the waveguide, resulting in the less confinement at the shrunken part of the waveguide. When the GST is completely crystalline, the transmission of the tapered structure (fig. S3A) is much larger than that in the first case. By comparing the two structures (Fig. 2C), we find that the electric field at the left edge of the crystalline GST film on the straight waveguide is high and gradually decreases to a very small value (E<10^6 V/m) at ~three fourths of the GST length. At this point, there is very limited transmission (5.04%) and substantial resonance at a period of 537 nm. However, for the tapered waveguide (S1), the electric field at the left edge of the crystalline GST is smaller and extends to the whole film. This does diminish at the resonant peaks (Fig. 2C) with a period of 544.8 nm in the right 20% region together with much stronger transmission (24.68%). Because of the poor confinement of the tapered waveguide, the interaction of the light with the GST is weaker resulting in higher optical transmission than the straight waveguide structure;

iii) To understand whether we can uniformly distribute the decay of the electric field along the propagation direction by discretizing the GST, we simulated the structure of S2 for the photonic synapse demonstrated in fig. S3B. In comparison with the continuous film of GST in the Standard structure, discrete crystalline GST islands allow a more uniform decay of the electric field that vanishes at the last GST island (E<10^6 V/m, Fig. 2C), resulting in more absorption and lower transmission (4.18%). The electric field resonance here is greatly attenuated compared to that in Standard and S1 designs (Fig. 2C).

iv) Finally, we simulated the structure combining the tapered waveguide and the discrete GST islands (Synapse-mimic design). Obviously, each crystal GST island has considerable electric field distribution (Fig. 2B) with larger absorption (i.e. resulting in slightly lower transmission, 18.18%) than the GST film in S1 structure. The resonance of electric field in Synapse-mimic design is further inhibited and rarely visible (Fig. 2C). In addition, the electric field distribution of the structures of S2 and Synapse-mimic are

further compared with different number of crystalline GST islands (0-6) (fig. S4). By increasing the number of crystalline GST islands, the electric field becomes inhomogeneously distributed in the GSTs for S2; however, the tapered waveguide structure (Synapse-mimic) shows a more even distribution of the electric field.

3. Photonic synapse switching mechanism

The novel structure of photonic synapse enables us to access a synaptic weight using the same pulses, and different from previous reports on memory applications (*23*). This is because:

i) Based on the FEM simulations in Fig. 2 and figs. S3 and 4, the tapered waveguide with discrete GST islands provides a smoother distribution of the optical field through the GST region that makes all GST islands respond to the optical pulse simultaneously to reach the same crystalline level;

ii) For a single GST island, the modulation from optical pulse trains results in a combination of amorphization and crystallization: the first pulse of a N pulse train will always result in the GST island being at the highest amorphous level (determined by the specific parameters of the single pulse) and the next N-1 pulses will bring the GST back to a less amorphous (more crystalline) state, depending on the value of N. This mechanism makes the final weight independent of the previous weights because it will always go to the highest amorphous level first;

iii) As all GST islands respond synchronously to the optical pulses, the synaptic weight will be at a determined value upon a fixed number of pulse trains.

4. Scalability, power consumption and signal-to-noise ratio

Scalability is a very important issue for on-chip neuromorphic photonic computing. Our manuscript focuses on a prototype demonstration of an on-chip photonic synapse and uses a number of off-chip components. However, there are obvious strategies to address system scalability and remove the need for off-chip components. For example, the circulators used in Fig. 1B to achieve the pump-probe scheme are not necessarily required. We can instead use a cross-bar waveguide to adjust the weight of the synapse via the vertical waveguide, while probing the synaptic weight (optical transmission) in the horizontal waveguide. Similarly, we used an interferometer to convert the time delay between pre- and post-spikes to pulse numbers in the proposed STDP design of Fig. 4. By incorporating on-chip interferometer with integrated phase control elements such as electro-optic or thermo-optic modulators, we can obtain a partial interference signal (from the pre- and post-spikes with phase compensation) to update the weight of the synapse, leading to integrated scalable circuits.

Turning now to the issue of the ***power consumption***, *P*, of synapses in a large scale network, this can be estimated simply by $P = (N \times E_s \times f)$, where *N* is the number of synapses in the network, *f* is the weighting update frequency and E_s is the energy required to update each synaptic weight. In our case, the energy E_s used for synaptic weighting is ~400 pJ for single pulse weighting and ~400 nJ when 1000 pulses are used. If we consider a large-scale system containing a billion synapses and updated at brain-like frequencies (say ~5 Hz), this results in a power consumption in the region of 2 W to 2 kW. Such powers are of the same order as for state-of-the-art electronic neuromorphic machines (e.g. SpiNNaker, which has a total overall power consumption of 80 W for a system of ~0.8 billion synapses and ~0.8 M neurons (*30*)) and less than that consumed by advanced neural processing carried out on conventional von-Neumann computers (e.g. the 2016 AlphaGo challenge, in which a computer beats for the first time a champion human GO player, using 1202 CPUs and 176 GPUs to implement a deep neural network and consuming ~40 kW of power (*34*)). Moreover, by using a combination of (i) picosecond optical pulses to switch the synapse (*32*), (ii) the replacement of the GST phase-change materials used in our work with compositions (e.g. Ge or N doped GST)

having lower switching powers and (iii) improved/optimized device design, it should, ultimately, be possible to reduce the total power consumption for a very large-scale photonic synaptic network down to a few tens of Watts, equivalent to that typically used by the human brain (*30*), but with a speed of operation orders of magnitude faster than that of the brain.

Finally we discuss briefly the topic of ***signal-to-noise ratio*** (SNR) in our synaptic devices. The noise in the output signal from the synapse is mainly from the photodetector and taking as an example the weight '0' (baseline) in Fig. 3C (and fig. S5), it has an amplitude (optical transmission) of 4.93×10^{-4}, while the noise amplitude (standard deviation) is 1.1×10^{-6}. This yields a SNR of ~450 (or 53 dB), which is already quite high but could be improved further by increasing the power of the probe laser (a strategy that has been demonstrated in our previous work (*23*)) and/or by using pulsed lasers, rather than the CW laser used here, to probe the synaptic weight (*32*). Such high SNRs bode well for integrating our photonic synapses into large-scale networks while retaining sufficient signal level for proper operation.

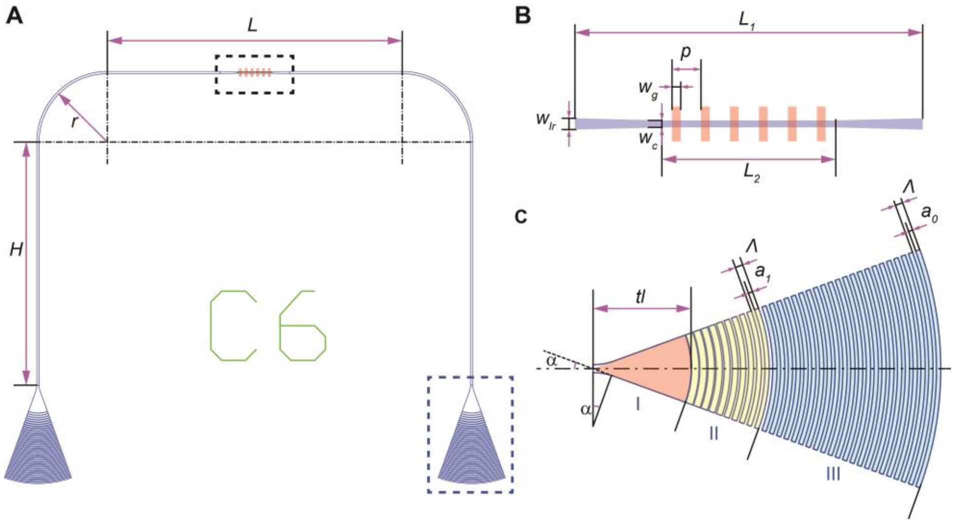

fig. S1. Design of photonic synapse. (**A**) Schematic of a photonic synapse structure. The major parameters are illustrated in the figure. (**B**) The zoomed-in region of the active device highlighted by black dashed box in (A), where the purple area is the waveguide and the orange rectangles are the PCM islands. The length of the central region is L_2 with a shrunk waveguide width of w_c. Several PCM islands (6 or 7) are equally distributed in the central region. The width of the waveguide linearly decreases from the outside (w_{lr}) to the central region (w_c). (**C**) The zoomed-in structure of the apodized grating coupler highlighted by the blue dashed box in (A) which has three regions: taper (I), apodized grating (II) and periodic grating (III). The grating period (w_g) of regions II and III is fixed at 1.27 μm with 10 and 22 grating periods in II and III respectively. The filling factor (a_0/w_g) in region III is 0.55 while that value is linearly increased to 0.98 in region II. All the details of the parameters are illustrated in table S1.

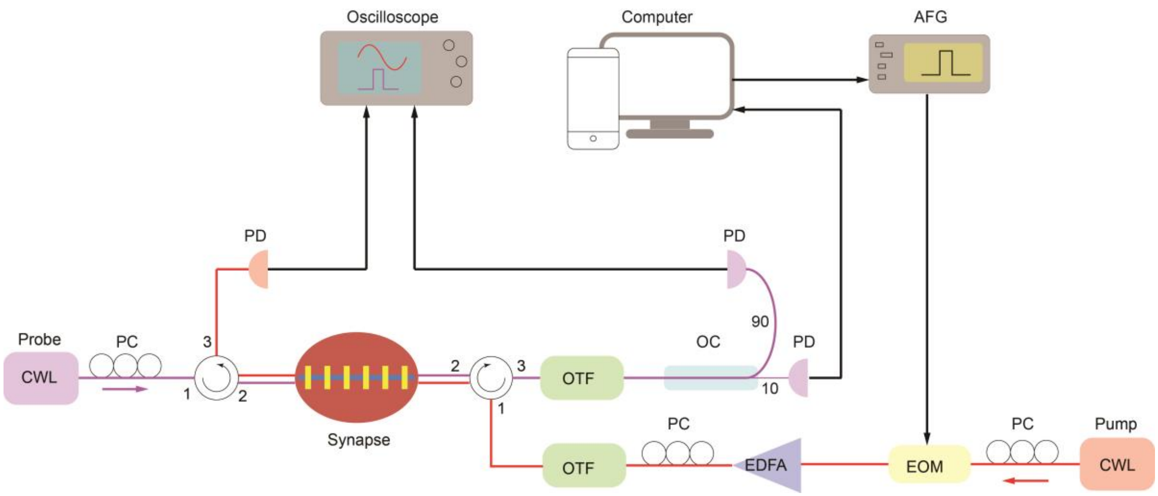

fig. S2. Optical measurement scheme. Schematic illustrating the probe/pump measurement strategy used to monitor the weight of the photonic synapse while switching the PCMs with optical pulses. Briefly, from left to right, the probe laser operates in CW mode and is guided in/out of the optical synapse by two circulators. It is subsequently filtered (OTF) and split (OC) into two beams detected by a slow 100 kHz (10) and a fast 125 MHz (90) photoreceiver (PD) connected to a computer and an oscilloscope respectively. The pump laser is pulse modulated (EOM), amplified (EDFA) and filtered (OTF) to obtain high power optical pulses and is detected using a fast (1 GHz) photoreceiver (PD) connected to the oscilloscope. CWL: continuous wave laser; PC: polarization control; OTF: optical tunable filter; OC: optical coupler; EDFA: erbium doped fiber amplifier; EOM: electro-optical modulator; PD: photo detector/receiver; AFG: arbitrary function generator.

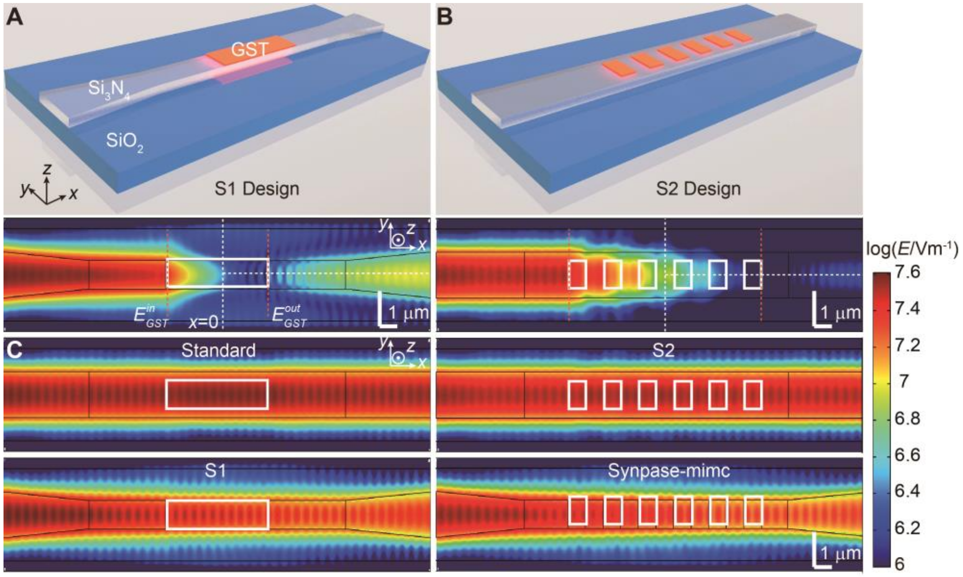

fig. S3. Optical field distribution in photonic synapses. (**A**) (Top) Schematic shows photonic synapse S1: the tapered waveguide structure with GST film kept the same as Standard design (the orange block) in Fig. 2A. (Bottom) TE mode electrical field distribution with the GST film (the white box) in crystalline state. (**B**) (Top) Schematic shows photonic synapse S2: the straight waveguide with six discrete GST islands (1 μm × 0.8 μm each, the orange blocks) on top. (Bottom) TE mode electrical field distribution with all GST islands (the white boxes) in crystalline state. (**C**) Electrical field distribution in the photonic synapses with all GSTs (white boxes) in the amorphous state. Standard, S1, S2 and Synapse-mimic designs correspond to Fig. 2A, fig. S3A, fig. S3B, and Fig. 2B respectively.

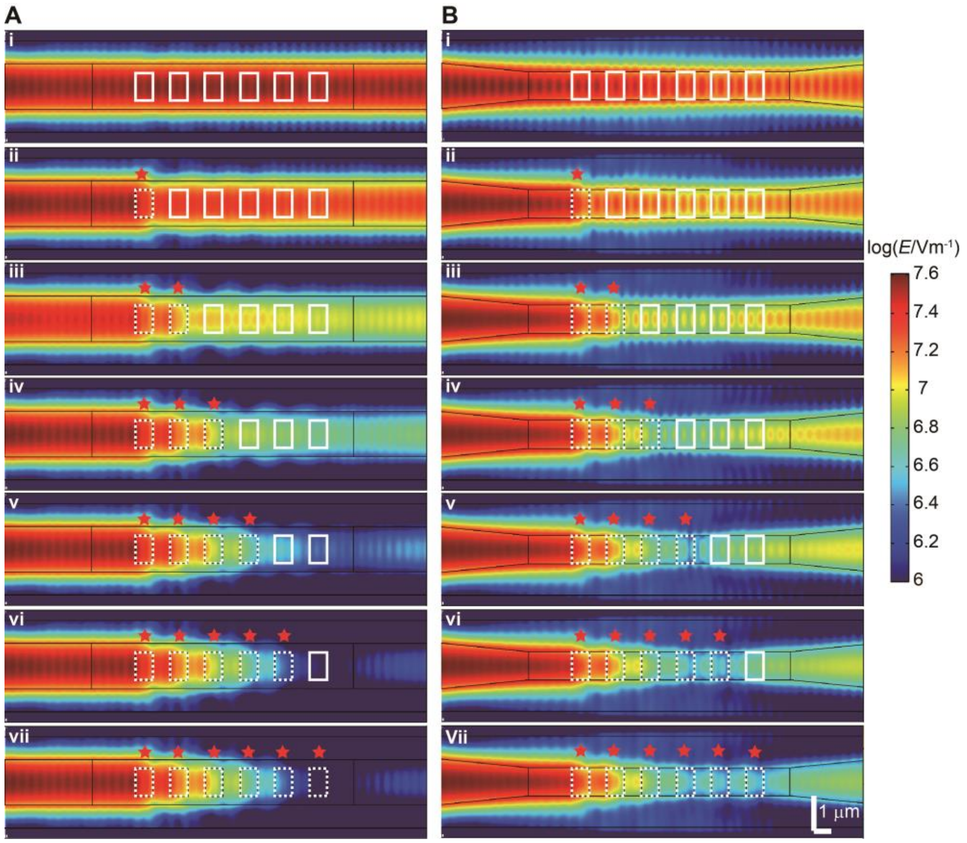

fig. S4. Optical field distributions in S2 and synapse-mimic designs. (**A**) Electric field distribution of TE mode inside the straight waveguide with six PCM islands on top. (**i** to **vii)**, different states of PCMs on the straight waveguide. Initially, all PCM islands are in the amorphous state (i, white box). Subsequently, 1 (ii), 2 (iii), 3 (iv), 4 (v), 5 (vi) and 6 (vii) islands are gradually crystallized (red stars and dashed white boxes). (**B**) Similarly, the electric filed distributions of TE mode in the tapered waveguide with the same number and parameters of PCMs in (A) are examined here. (**i** to **vii**) different states of PCMs on the conventional waveguide. All PCMs are in the amorphous state (i). Afterwards, 1 (ii), 2 (iii), 3 (iv), 4 (v), 5 (vi) and 6 (vii) pieces are gradually crystallized (red stars and dashed white boxes).

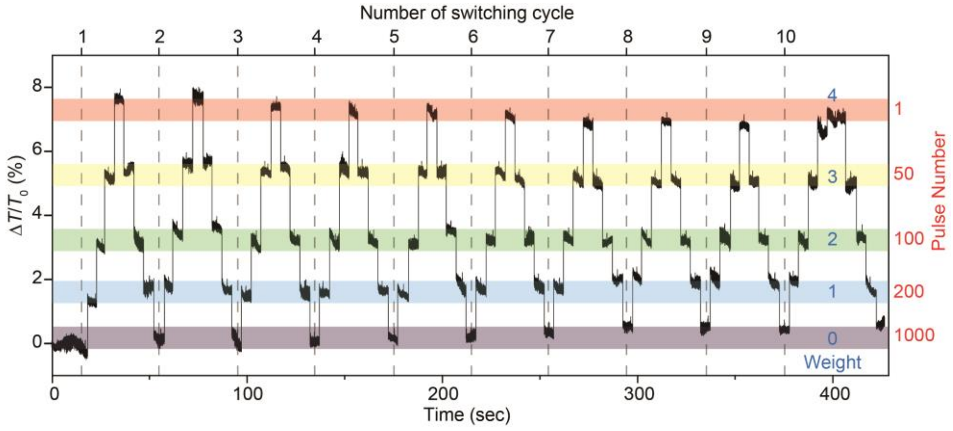

fig. S5. Full trace of five-level weighting. The full trace of the weighting in Fig. 3C in the main text shows five weights that are clearly distinguished. The device is #1 with dimensions illustrated in table S1. The five weights can be reached as long as 7 mins switching with 10 cycles and each level corresponds to a pre-determined number of pulses (404.5 pJ, 50 ns): 1, 50, 100, 200 and 1000.

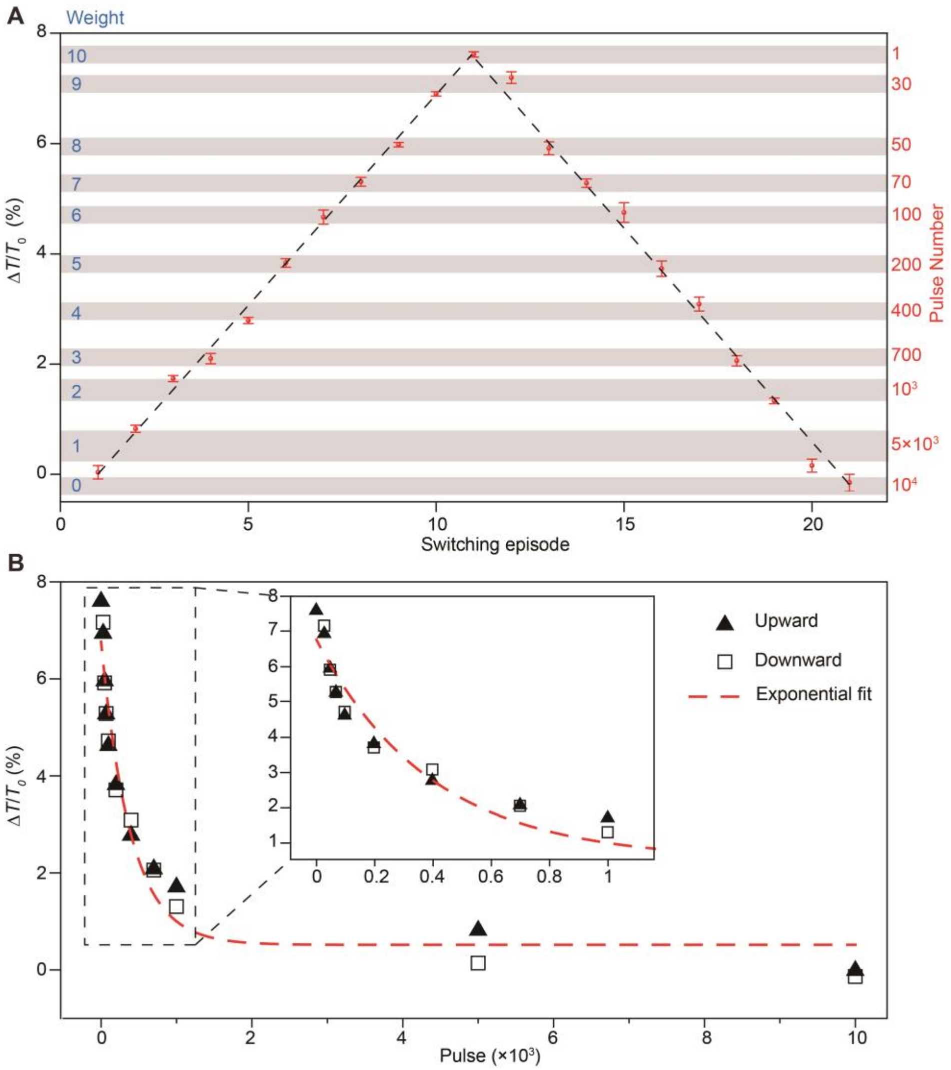

fig. S6. Eleven-level weighting. (**A**) Optical transmission change of sample #2 shows 11 weights with pre-determined numbers of 20 ns optical pulse (216 pJ) with upward and downward sweeps. The parameters of sample #2 are elucidated in table S1. The corresponding number of pulses for each level is illustrated in the figure. (**B**) The dependence of the transmission change on the pulse number. The solid triangles and hollow squares correspond to the upward and downward weighting of the synapse in (A) respectively. The fitting parameters are shown in table S2. The inset shows the zoomed-in region of the pulse number between 1 and 1000, highlighted as the dashed box.

table S1. Dimensions of photonic synapses. Sample #1 has six islands of GST on top of the waveguide that is used for Figs. 1 to 3 in the main text and fig. S3 in Supplementary Materials. Sample #2 with seven pieces of GST is used for fig. S6. The parameters for the apodized grating coupler are optimized with the resonant wavelength at 1580 nm.

**No.**	L (μm)	H (μm)	r (μm)	L_1 (μm)	L_2 (μm)	w_{lr} (μm)	w_c (μm)
**#1**	170	100	40	40	20	1.3	0.8
**#2**	170	100	40	35	15	1.3	0.8
	w_p (μm)	p (μm)	tl (μm)	Λ (μm)	a_0/Λ	a_1/Λ	α (°)
**#1**	1.0	3.33	16	1.27	0.55	0.55~0.98	40
**#2**	0.75	2.14	16	1.27	0.55	0.55~0.98	40

table S2. Fitting of synaptic weight on pulse number. The pulse number dependence of synaptic weight in Fig. 3D and fig. S6B are exponential fitted with parameters shown in the table. Here ΔT is the synaptic change or relative transmission change and N_p is the pulse number. *std* means standard deviation.

**Fitting Model** and **Parameters**		**Fig. 3D**		**fig. S6B**
$\Delta T = \Delta T_0 + A \times exp(B \times N_p)$		Left panel	Right panel	
ΔT_0	Mean	0.0819	0.516	5.17×10^{-3}
	std	0.176	0.104	2.65×10^{-3}
**A**	Mean	7.72	6.65	0.0626
	std	0.276	0.162	3.3×10^{-3}
**B**	Mean	-8.65×10^{-3}	-8.28×10^{-3}	-2.55×10^{-3}
	std	6.47×10^{-4}	4.23×10^{-4}	4.12×10^{-4}
**Adjust R-Square**		0.99	0.995	0.945
